# Analysis of Marine Microplastic Pollution of Disposable Masks under COVID-19 Epidemic—A DPSIR Framework

**DOI:** 10.3390/ijerph192316299

**Published:** 2022-12-05

**Authors:** Ge Song, Hu Cao, Lanyi Liu, Min Jin

**Affiliations:** School of Environment and Natural Resources, Renmin University of China, No. 59 Zhongguancun Street, Beijing 100872, China

**Keywords:** marine microplastic pollution (MMP), disposable masks (DMs), COVID-19 pandemic, DPSIR framework

## Abstract

Marine microplastic pollution (MMP) is becoming one of the most pressing environmental problems facing humanity today. The novel coronavirus epidemic has raised the issue of environmental contamination caused by large-scale improper disposal of medical waste such as disposable masks (DMs). To assess the impact of MMP caused by DMs and to seek solutions for the prevention and control of MMP, this study uses the Driving force-Pressure-State-Impact-Response (DPSIR) framework to establish a causal chain of MMP caused by DMs. The conclusion shows that the novel coronavirus epidemic has led to a surge in the use of DMs, which has brought pressure on resource constraints and environmental pollution at the same time. Improperly DMs enter the environment and eventually transform into MMP, which not only endangers the marine ecological system but also poses potential human health risks as well as economic and social hazards. In addition, further research on environmentally friendly masks (cloth masks and biodegradable masks) is essential to mitigate the environmental damage caused by the large-scale global use of DMs. This study provides a scientific and theoretical basis for the assessment of MMP from discarded DMs, and the findings of this study will provide a reference for the formulation of relevant policies.

## 1. Introduction

The emergence of the novel coronavirus (COVID-19) disease with strong contagiousness has attracted global attention since December 2019 [1]. The extensive use of face masks among health workers and the general public worldwide is regarded as an effective way to stop massive infections [2]. Some countries have successfully brought their epidemics under control using mask policies, such as China and Italy [3,4]. Since 2021, the more contagious Delta and Omicron variants of COVID-19 have been spreading severely around the world [5]. With the lifting of the lockdown restrictions and the resumption of global trade, a growing number of countries are recommending that citizens wear masks in public places [6], which causes a sharp increase in the demand for disposable masks (DMs) [7]. This situation has resulted in the increasingly prominent problem of mask waste disposal. 

DMs are mainly made of polypropylene (PP), which is considered plastic waste after being discarded. Its entry into the environment can cause plastic pollution and release microplastics, severely threatening the aquatic environment and aquatic life once they enter freshwater and coastal areas [8]. The data show that from 21 January 2020 to 19 March 2021, approximately 6.5% to 11.9% of the total amount of plastic entering the oceans worldwide was derived from DMs, which is a significant figure [7]. In the post-pandemic era, DMs should be of concern as a new source of contamination of environmental microplastic fiber. There is an urgent need to recognize this potential environmental threat and prevent it from becoming the next microplastic problem [9].

Several studies have focused on the MMP induced by the rapid growth of waste in DMs. Sangkham measured the generation of DMs and medical waste in Asia during the outbreak and discussed the emerging challenges in solid waste management during and after the pandemic [10]. Jung et al. proposed a technical solution for environmentally sound recycling and treatment of disposed DMs to reduce the possibility of them entering the environment, especially the water and ocean [11]. By comprehensively assessing the ecosystem impacts of COVID-19-related plastic wastes, particularly discarded DMs, Kutralam et al. have demonstrated that the widespread prevalence of these plastic wastes has exacerbated microplastic pollution in oceans and rivers [12]. Silva et al. discussed the evolution of plastic waste policies worldwide since the epidemic of COVID-19 and made corresponding policy recommendations for improving waste management [13]. However, the problem of MMP from discarded DMs cannot be addressed from a single perspective but needs to be viewed holistically and sustainably. Existing studies do not adequately recognize the complexity of the system and lack an integrated understanding of the interactions between society, the economy, and the environment in the MMP problem from discarded DMs.

In order to fill this gap, this study analyzes and explores the problem of MMP caused by DMs from a multidimensional perspective, including social, economic, and environmental perspectives. The structured approach provided by the DPSIR framework is used to examine, in detail, the interrelations among the five components to better understand the current complex pollution situation of marine microplastics and the potentially significant pollution risks in the future. First, the driving forces of DM usage are discussed. Second, the social and environmental pressures created to meet human demand are examined. Then, the physical, chemical, and quantity states of the DM contaminants and the impact of their resulting impacts on marine ecosystems, human health and socioeconomics are examined. Next, responses to regulate the driving forces, mitigate the pressures and states, and impacts of MMP are reviewed from both upstream and downstream perspectives. Finally, specific recommendations for preventing and controlling MMP from DMs are derived.

## 2. Methodology

MMP is a complex environmental problem that requires an adaptive management framework that is action-oriented, integrated with an approach which is interdisciplinary in nature, incorporating both social and ecological systems [14]. The DPSIR framework was first developed by the European Environment Agency (EEA) in 1995 as a conceptual framework for looking at environmental issues from environmental, economic and social perspectives [15]. The framework is considered an important environmental assessment tool for developing and maintaining appropriate and adaptive management responses [16], which analyzes various environmental issues by identifying causal relationships between human activities and relevant environmental and socioeconomic outcomes [17,18]. In fact, the DPSIR framework is considered useful to identify the driving forces of environmental damage and the associated policy and regulatory responses [19].

The DPSIR framework is one of the key conceptual frameworks that are now widely used in marine environmental issues. It has been applied to various marine environmental issues, such as social-ecological systems in coastal environments [16], social risks of marine pollution [20], and constructing coastal and marine ecosystem management approaches [21]. The framework can convey the complex interactions between society, the economy, and the environment. It not only provides policymakers with a basis for policy formulation but also raises public awareness of policy measures.

### 2.1. Concept of DPSIR Framework

**Figure 1:**
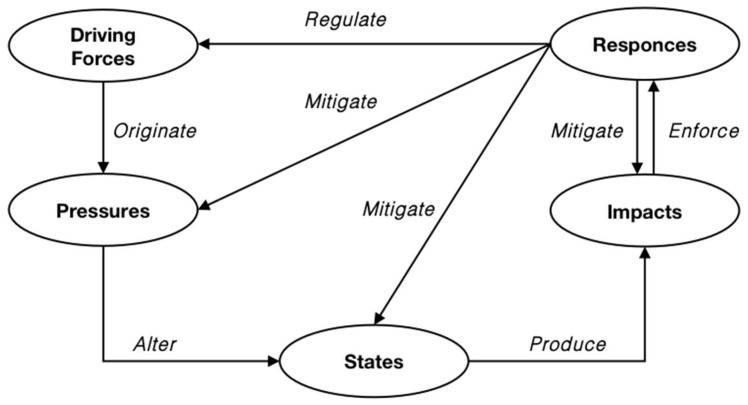
The general framework of DPSIR.

According to the EEA [22], the components of the DPSIR framework are defined as: (a) Driving forces are societal changes that shape demand, consumption, and consumption patterns that put pressure on the environment; (b) Pressures are emissions and resources deployed to meet driving forces; (c) States describe the state of physical, chemical and biological properties in terms of quantity and quality; (d) Impacts are changes in ecosystem function and negative impacts on human health and economic activities, such as loss of biodiversity, income, and well-being; (e) Responses include social and government initiatives to prevent, mitigate and adapt to the changes. Figure 1. shows the general framework of DPSIR.

With the widespread use of the DPSIR framework in environmental studies, the definition of the five components of the framework and the relationships among them have been explained in more detail.

(1) The “driving forces” are the most primitive factors that cause environmental change and refer to the social, economic and institutional changes that directly or indirectly trigger environmental stress. It can be defined as “social, demographic, socio-economic development and corresponding changes in lifestyles, as well as overall consumption levels and production patterns” [23]. Rodriguez [24] has divided the level of driving forces into four levels, including “primary driving forces”, “secondary driving forces”, “tertiary driving forces” and “basic driving forces”, which correspond to “socio-economic activities directly related to pressures on the natural environment”, “relevant policies and laws and regulations”, “ideologies and lifestyles” and “trends influenced only by long-term social decisions, such as demographics, culture, etc.”
(2) The “pressures” refer to anthropogenic factors that are directly applied to environmental systems by driving forces and contribute to environmental change, “including emissions of material, physical and biological agents, and the use of resources and space by human activities” [23]. Pressures reflect changes, levels of degradation, and trends in the environment.
(3) The “states” indicate both qualitative and quantitative measurements of pollutants or the current state of the environment or environmental parameters that may change due to pressures [23]. It is worth mentioning that state factors can be explored from the micro level of the physical and chemical state of the pollutant itself [6], or from the macro level of the integrated system composed of pollution sources and environmental composition. Depending on the point of concern, state indicators may vary considerably.
(4) The “impacts” refer to the feedback effects of natural system states on human health, socioeconomics, and ecosystems, which often arise from adverse environmental conditions and are the result of a combination of the first three elements [23]. Impacts reflect changes in ecosystem quality and functions that affect human and social well-being.
(5) The “responses” refer to social actions taken by humans to prevent, mitigate, or eliminate negative impacts [23], which in turn have some impact on the trends of the first four elements.

### 2.2. Using the DPSIR Framework to Analyze the MMP from DMs

**Figure 2:**
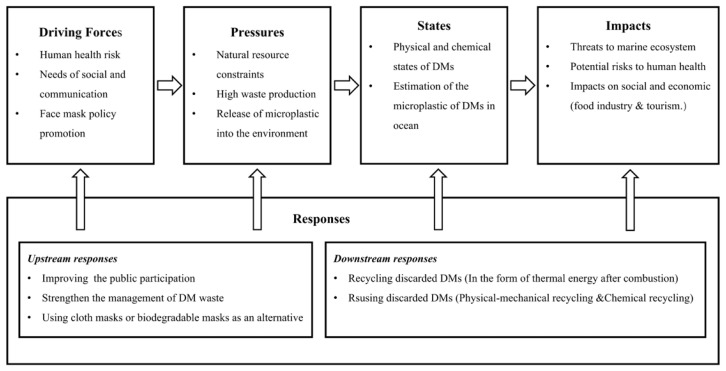
The DPSIR framework for analysis of disposable masks in marine microplastic pollution.

The DPSIR framework is used to address the MMP from DMs, following a typical five-step procedure (Figure 2). In this study, the framework is appropriately adapted and improved according to the salient features of the research problem. (a) The main sources of the driving forces are anthropogenic and natural factors [25]. In the study of DMs as a source of microplastic pollution, the focus was on MMP from anthropogenic factors. Three aspects of human health risk, needs for social and communication, and mask policy promotion are identified as driving forces in this study. (b) The pressures are mainly in the areas of population growth, resource shortage, and environmental damage. In the study of microplastic pollution of DMs, the pressure factors come from three components: natural resource constraints, high waste production, and the release of microplastics into the environment. (c) As a source of microplastic contamination, the physical and chemical state of the DM itself determines the resulting environmental impact. The quantity of microplastics in the ocean from DMs reflects the degree of contamination of the marine environment influenced by pressure factors. (d) This study focuses on the impacts of MMP caused by discarded DMs on marine ecosystems, human health, as well as society and the economy.

## 3. Analysis of DMs in MMP Based on the DPSIR Framework

### 3.1. Driving Forces

**Figure 3:**
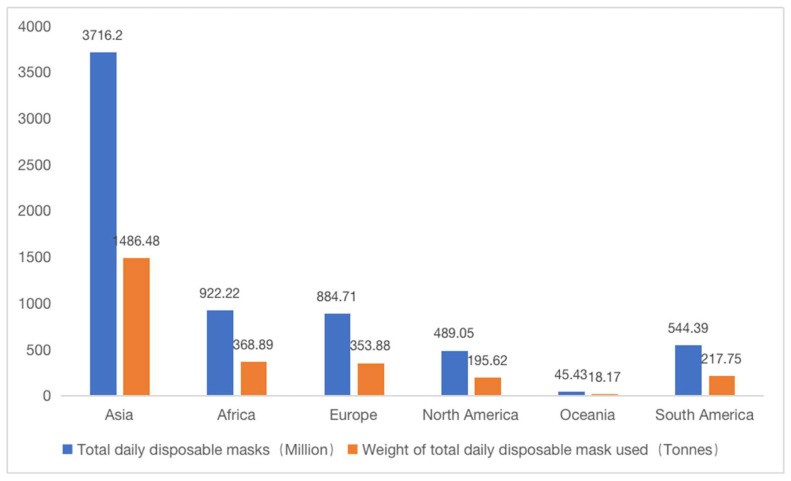
The estimated number of DMs used during the pandemic [30].

The COVID-19 pandemic has severely impacted the health and livelihood of people worldwide. DMs have a great filtering effect on aerosols and droplets, which can prevent respiratory diseases. Wearing DMs effectively reduces the probability of being infected by novel coronavirus [26], which means that they are not only essential protective items for health care workers but also essential for the public to avoid COVID-19 [27]. As a result, the global demand for DMs has increased approximately 100 times since the outbreak of COVID-19 compared to the pre-epidemic period [28]. The global mask market increased its value from USD 790 million in 2019 to approximately USD 166 billion by 2020 [29]. Figure 3 shows the estimated number of DMs used during the pandemic.

To avoid the crisis of economic downturn and communication blockage brought about by precautionary measures such as lockdowns and travel restrictions, people in different countries choose to wear DMs on a large scale, while maintaining social distancing, keeping good hand hygiene (hand washing), and avoiding public or crowded spaces as alternative measures to keep society functioning properly [31].

Since 2020, health authorities in different countries have issued various recommendations for using face masks to protect against COVID-19, which tend to be dynamically adjusted (Table 1) [32]. While some health agencies in the early days of the COVID-19 outbreak believed that wearing masks had a limited effect on protecting individuals (non-patients and health care workers) from infection, recently, health agencies from these countries have coincidentally emphasized the importance of wearing masks in daily use scenarios. For example, Japan has made it mandatory to wear a DM when meeting the elderly. The United States has abolished the compulsory wearing of face masks on public transportation in favor of recommendations. Germany has mandated the use of the DMs with a specific high protection level for local and long-distance public transport. China has implemented the most stringent mask policies, detailing the types of masks used for different scenarios and groups of people. Mandatory requirements for using face masks are the same in all countries for people with respiratory symptoms or caring for someone with symptoms. Recommendations for masks vary from country to country, and it has been seen that once the local epidemic began, the use of masks increased dramatically [33].

### 3.2. Pressures

The raw materials of DMs are mostly made of petroleum-based polymer-PP (PP) fiber, which is a non-renewable resource. Therefore, the massive use of DMs can cause a large consumption of natural resources. Furthermore, DMs are used in a wide range of applications and scenarios. They are likely to be directly discarded in municipal solid waste without safe disposal measures or directly in the environment. This creates new challenges for medical waste management, which will worsen especially for developing countries with inadequate waste management [42]. 

Discarded DMs are found scattered in public places such as parks and streets all over the world [33]. A study from Australia showed that discarded DMs could easily be shredded into melt-blown fabric fragments when affected by lawn-cutting equipment. After the crushing and grinding process, they were mixed into the soil medium, resulting in a dispersion of spatial distribution [43]. A study of the potential of DMs to release microplastics into the environment showed that the average microplastic release from unused DMs ranged from 71.7 to 308.3 units per piece, increasing from 682.7 to 1918 units per piece after use. Based on the global monthly usage of 129 billion DMs [44], the daily microplastic release from used DMs is more than 29,000 billion. It is estimated that approximately 1.56 billion DMs entered the oceans globally in 2020, resulting in between 4680 and 6240 metric tons of marine plastic pollution. It will take up to 450 years for these discarded DMs in the ocean directly or indirectly to break down and slowly turn into microplastics [42]. The source of microplastics is expected to continue to increase in the coming years due to the overuse of DMs to fight COVID-19.

### 3.3. States

#### 3.3.1. Physical and Chemical States

The DMs are made of melt-blown fabric, non-woven fabric, mask straps, and nose clips. Melt-blown fabric is the core material used to produce DMs, whose main component is PP. The melt-blown cloth consists of ultra-fine fibers with diameters of about 1–5 microns arranged randomly to form numerous tiny pores that trap dust due to the electrostatic effects. When the droplets containing various types of viruses approach the PP melt-blown fabric, they can be adsorbed on the surface of the melt-blown fabric and cannot penetrate. Thus, DM has a strong filtration property, sufficient to reject bacteria, suspended particles, liquid droplets, and aerosols. The inner layer, near the mouth and nose, is made of hot-rolled nonwoven fabric to absorb moisture and ensure that the wearer’s lips are dry and comfortable. The entire mask is designed to fold to improve its fit to the wearer’s face, blocking more than 90% of the 5μm particles. The structure of DMs varies slightly between standards, with disposable surgical masks and N95 masks having a high acceptance rate among health workers and the public [45]. Their structures are shown in Table 2.

Experimental results show that individual N95 masks and disposable surgical masks have approximately 11 and 4.5 g of PP and other plastic derivatives (e.g., polyethylene, polyurethane, polystyrene, polycarbonate, polyacrylonitrile), respectively [46,47]. Because of their multiple drug resistance, hydrophobic properties, high molecular weight, and high surface roughness, DMs often do not completely degradade and mineralize [48]. As a result, most PP remains in the environment in the form of microplastics, most of which eventually disperse into the oceans.

The use of DMs has reduced the spread of the virus in society while simultaneously causing severe environmental problems in terms of microplastic pollution. During the use of DMs, extremely fine nonwoven fibers tend to break off and form microplastics. Mechanical wear and tear between the fibers is further exacerbated by the repeated use of DMs over time, leading to extra microplastics. In addition, DMs may intercept microplastics in the air during use, thus increasing their microplastic carrying capacity [49]. 

#### 3.3.2. Estimation of the Number of Microplastics of DMs in Ocean

Discarded DMs enter the ocean through improper landfills and dumping treatment. They disintegrate into tiny plastic fragments under a range of physical and chemical effects, such as solar radiation, biological erosion, low-temperature weathering, tidal, wave washing, and so on. As the DMs gradually age and decompose in the environment, the entire DM will become completely microplastic and enter the ocean [49]. Shen et al. have experimentally found that when a DM is naturally aged in the environment for 2 months, it can release billions of microfibers into the marine environment [49]. Similar results were obtained by Aragaw, where the number of microplastics released from a used DM was 6.0–8.1 times higher than the number of microplastics released from a new one [50]. In an experiment simulating the marine environment, Saliu et al. concluded that a single surgical mask could release 17,300 units of microplastic fibers in 180 h [51]. DMs discarded in the ocean can completely break down into microfiber fragments and aggregates within two years. It is estimated that in 2020, 3.5 million tons of DM waste have been landfilled globally and these DMs have the potential to release 2.3×10^21^ units of microplastic into the environment [52]. Using kinetic studies, it has been estimated that discarded DMs in 2020 have resulted in over 1370 trillion microplastic entering the global marine environment, with a daily release rate of 39.6 billion microplastics [53]. The available evidence suggests that microplastics from discarded DMs are a significant source of MMP. 

### 3.4. Impacts

Unlike other plastic products such as plastic bags and bottles, DMs are more likely to be damaged and decomposed in the natural environment, releasing additional microplastic fibers [54]. The melt-blown fabric in the middle layer of the DMs is susceptible to UV exposure. A single weathered mask can release more than 1.5 million microplastics into the aqueous environment. Physical abrasion caused by sand further exacerbates the release of microplastic particles from the DMs [55]. Moreover, DMs release microplastics more readily and rapidly in nature than plastic bags and boxes. They are a easily overlooked and potential source of marine microplastics, with long-lasting negative impacts on marine ecosystems, which in turn pose risks to human health and safety and create social and economic risks [56].

#### 3.4.1. Threats on Marine Ecosystems

Discarded DMs entering the environment may not only entangle the bodies of marine wildlife but also release microplastics that are preyed upon by marine life, thus affecting normal growth and reproduction [57]. Experimental studies have shown that microplastic particles can be ingested by aquatic organisms and cause physical hazards to them, such as blocking their feeding aids and digestive tracts, producing pseudo-satiety, depleting the stored energy of organisms, crossing cell membranes, and affecting the enzymatic activity of cells [58]. Microplastics bring physical pollution as well as compound pollution. Due to the feature of small size, slow degradation rate, strong adsorption capacity [59], and easy migration [60], microplastics tend to be suitable carriers of organic pollutants and heavy metal pollutants to flow in the environment, thus creating a continuous hazard. 

Microplastics have been found in organisms at different levels of the marine food chain [61,62]. The results of the survey showed that among the investigated wildlife species, the species of sea turtles, marine mammals, and seabirds affected by plastics (including microplastics) increased from 86%, 43%, and 44% in 1997 to 100%, 66% and 50%, respectively, in 2015 [63]. Thus, MMP has become an essential threat to marine ecosystems [64,65,66].

#### 3.4.2. Potential Risks on Human Health 

With the increasing amount of marine organisms caught and seafood supplied by aquaculture year by year, microplastic particles in these marine organisms and aquaculture products may eventually enter the human food chain. The pollution status of the marine environment by microplastic is a potential health problem for humans. It has been shown that harmful microorganisms could attach to plastic debris [67], increasing the risk of pathogen transfer and disease development. Moreover, microplastics from marine organisms are easily ingested by humans through the food chain due to food chain transfer and bioconcentration, posing a potential risk to human health, which may lead to many adverse effects such as cancer, reduced immunity, impaired cellular activity, and disruption of the gut microbial community [68]. It has been confirmed that polystyrene with different particle sizes has different toxic effects on gastrointestinal cells [68]. Hwang et al. [69] found that direct contact between microplastic particles and cells could induce cytokine production by immune cells and cause a series of health problems by studying the hazard of PP microplastics in human-derived cells. However, it is difficult to assess the presence of microplastics in the human body and their effects on the human body at this stage. At this stage, researchers have found the presence of microplastics in the marine environment [70], soil environment [71], domestic water [72], salt [72], and even maternal placenta [73], and their presence should not be underestimated.

#### 3.4.3. Impacts on the Society and Economy

Foods made from marine organisms, such as fish, are an important food source for humans. MMP raises concerns about global food security, which may eventually lead to the consequences of food shortages [74]. In the tourism industry, the ecology of coastal areas is very important for the development of tourism. However, once the coastal environment is contaminated with discarded DMs, thus reducing the aesthetic, recreational value and tourism experience, it will greatly reduce the economic benefits of the tourism industry [75].

### 3.5. Responses 

To respond to the impact of MMP from DMs, especially the impact on marine ecosystems, the potential impact on human health, and the impact on the economy and society, a series of prevention and control strategies have been developed at different levels. According to the principles of source reduction and process management of MMP prevention and control [76], the response to MMP from DMs can be divided into two parts: upstream response and downstream response. 

#### 3.5.1. Upstream Responses

In the context of the normalization of epidemic prevention and control, DMs have become an immediate need in people’s daily lives, and their demand cannot be reduced in the short term [77]. The fundamental measure of source production and consumption reduction has limited space to be carried out. Therefore, the upstream response measures should be mainly from improving public participation in the proper disposal of DMs, strengthening the management of DM waste, and developing environmentally friendly mask alternatives.

(1) Improving public participation in the proper disposal of DMs

Guiding the public to properly dispose of DMs is the most effective way to properly prevent the problem of microplastic pollution at the source. Used DMs can greatly reduce the risk of environmental contamination after proper disposal such as disinfection and incineration [44,78]. At present, the improper disposal of DMs by the public is the main source of DM waste in the environment, and this kind of behavior is difficult to regulate. It requires certain guidance and incentives to guide the public to pay attention to the scientific discarding of DMs, the popularization of the concept of consumption, the change of discarding habits, and the formation of the behavior of separated waste disposal. The means of publicity and education need to be emphasized. Use new media and other means to inform and educate the public, especially in less developed countries and regions [45]. Taiwan has introduced regulations to punish citizens who do not dispose of DMs as required and implemented financial rewards for reporting mask litterers [79].

(2) Strengthen the management of DM waste

Due to the disposable natures of DMs, recycling as a reduction pathway to achieve more limited results, and scientific management based on the disposal process will be sought to reduce the risk of MMP caused by DMs. The COVID-19 pandemic has increased the pressure on municipal waste management, which can lead not only to the spread of the virus but also to environmental problems if not handled properly [10]. Therefore, it is necessary to plan and consider separation, storage and collection for recycling and disposal of DMs and medical waste to reduce plastic waste. Collection sites should be established in streets and other public places, and citizens should be encouraged to put DMs in specific bins instead of putting them in regular garbage cans or carelessly discarding them [7].

Currently, several medical waste bins with clearly marked points are placed in public areas such as hospitals and communities to collect discarded DMs. They are packaged in double-layered medical waste bags and disposed of as general medical waste by specific personnel, municipal solid waste workers, and special waste management departments of companies [80]. 

(3) Using cloth masks or biodegradable masks as an alternative

The promotion of cloth masks can effectively reduce the consumption of DMs [6,45]. Cloth masks with filtration capabilities ranging from 60% to 95% are effective against respiratory droplets, bacteria, and particles [81,82]. It has been demonstrated that cloth masks have similar protection efficiency to DMs in air-conditioned microenvironments [83]. In a comparison of the environmental impact of the two types of masks in different situations, it was found that the cloth masks produced 85% less waste than their disposable counterparts, with a definite advantage in reducing waste plastic output in particular [6]. Centers for disease control and prevention (CDC) guidelines focused on the use of cloth masks as personal protective equipment and directed the public to make their own cloth masks [84]. Although current studies on the cost-benefit analysis of cloth masks are insufficient, based on available studies, cloth mask materials such as cotton, gauze, silk, or muslin are accessible and reusable. Therefore, it has a lower cost of use and environmental pollution impact compared to DMs in the long run [85]. Therefore, the next step of research may follow the development of cloth masks as an environmentally friendly alternative to DMs [83].

The development of biodegradable masks is another solution idea to reduce the risk of the DMs contamination. The advantage of biodegradable materials is their biodegradability through composting, which avoids environmental hazards at the source to some extent. The biodegradable polymer and bio-based material electrospun ensemble can be used as the main material of masks. Experiments revealed that this type of mask reaches 98% particle filtration efficiency during use, and its waste can be biodegraded within four weeks [86]. In addition, the application of some plant fiber materials in mask production offers the possibility of moving masks to zero waste [87]. It has been demonstrated that biodegradable masks have better environmental and economic benefits than DMs in terms of energy savings, pollution reduction and lower carbon footprint from production, transportation and use. More and more biopolymers are being used as materials for masks, providing the public with a wide range of choices for masks that are environmentally friendly, comfortable, and affordable [88]. Future development research can make efforts in assessing the effectiveness and longevity of biodegradable masks.

#### 3.5.2. Downstream Responses

The downstream responses mainly focus on recycling and reusing discarded DMs to reduce the risk of MMP. Medical plastic waste generated during the epidemic has a high calorific value compared to other conventional fuels, accounting for nearly 25% of the calorific value of municipal solid waste [89]. Discarded DMs can be recovered in the form of thermal energy after combustion, but the use of recovered thermal energy remains an obstacle, and trace emissions of products of incomplete combustion (PIC), dioxins and furans cause additional environmental pollution. Mechanical recycling, and chemical recycling methods have been used to extract PP from discarded DMs and convert the waste into various end products to achieve resource conservation and pollution reduction.

(1) Physical-mechanical recycling

Improving the process of mechanical recycling of DMs can produce the recycled product, recycled polyethylene, with better stiffness and strength, which enables physical and mechanical recycling [90,91]. The physically cut fiber fragments of the discarded DMs can be mixed into the recycled concrete aggregate (RCA) to enhance the ductility and durability of the concrete. It is predicted that approximately 3 million DMs can be consumed to build a 1 km long two-lane highway using this method, effectively solving the problem of disposal of the DM [92]. In addition, after being fully disinfected, discarded DMs can also become raw materials for wood-plastic composites and wood-plastic composite mulch, which can effectively reduce environmental pollution.

(2) Chemical recycling

Multi-stage pyrolysis was used to catalyze the cracking of discarded DMs to generate H_2_, CH_4_, and CO, which suggests an environmentally benign disposal process [11,93]. Xiang Hu et al. used a high temperature and high-pressure reaction kettle to carbonize discarded DMs to obtain hollow fiber porous structure raw materials for the preparation of high-performance supercapacitor electrode materials with high added value and environmental protection [94]. Therefore, chemical recycling is also an effective measure to reduce the ecological impact caused by discarded DMs.

## 4. Conclusions

DMs, as a new type of microplastic pollutant, can continuously release microplastics into the marine environment through the water systems. However, the rigidity of their demand and single-use nature makes it difficult to establish closed-loop chain management of the entire life cycle such as source reduction, waste process control, and end-of-life recycling. This study is based on the analytical framework of DPSIR, which seeks more effective management measures to curb the MMP prevention and control brought by discarded DMs. The results of the study show the surge in demand for DMs’ uses under the three driving forces of human health risk, social demand, and mask policy promotion in the context of the COVID-19 epidemic, increased the risk of non-renewable resources such as oil tension, plastic waste increase, and microplastic flow into the environment, thus increasing the pressure on both resources and the environment. In addition, the physical-chemical composition of discarded DMs leads to waste ending up in the ocean in the form of large amounts of microplastics. In order to effectively reduce the impact on the marine ecosystem, human health, and socioeconomics caused by MMP from DMs, response mechanisms are proposed for upstream and downstream in a targeted manner.

In view of this, countries around the world should guide the public on the proper disposal of DMs and educate the public on the environmental responsibilities that individuals should assume. Secondly, it is necessary to strengthen the management of DM waste and improve the infrastructure to help the public dispose of DMs reasonably. In addition, a system of rewards and penalties for recycling DMs could be developed as a complement to fully incentivize the public to dispose of DMs reasonably. Moreover, eco-friendly alternatives such as cloth masks and biodegradable masks with filtration efficiency that meet basic protection needs and have a longer life cycle can be developed. In addition, further research into the risks of MMP from DMs is essential, and new technologies should be developed to overcome the problems associated with the release of microplastics from DMs.

## Figures and Tables

**Table 1:** Mask use recommendations by different health authorities.

Countries/ Regions	Main Points
WHO [34]	Make wearing a mask a normal part of being around other people. The appropriate use, storage and cleaning or disposal of masks are essential to make them as effective as possible.
USA [35]	On January 29, 2021, the Centers for Disease Control (CDC) order requiring masks on public transportation conveyances and at transportation hubs. On April 18, 2022, CDC did not enforce the order but continued to recommend that people wear masks in indoor public transportation settings at this time.
China [36]	The N95 respirator and the surgical protective mask are required by infected patients, staff working for patients, emergency department staff; public health physicians who conduct epidemiological investigations, and environmental and biological sample testers. The surgical mask is required by medical personnel, staff in crowded places, personnel related to the epidemic, and people quarantined at home. The single-use medical face mask is required by the public in crowded areas (such as supermarkets; shopping malls; transportation, and elevators), indoor office environments, patients treated by medical institutions (except for fever clinics), children in kindergarten and school students who intensively study and have activities, etc.
Japan [37]	It is important to wear a mask as a basic prevention measure against COVID-19. Wear a mask in crowded areas (e.g., public transit). Wear a mask when you meet with elderly or spend time in a hospital. If you are outdoors and not talking to others, remove your mask to prevent summer heat stroke.
Hong Kong [38]	Surgical masks can prevent transmission of respiratory viruses from people who are ill. It is essential for people who are symptomatic (even if they have mild symptoms) to wear a surgical mask. Wear a surgical mask when taking public transport or staying in crowded places. It is important to wear a mask properly and practice good hand hygiene before wearing and after removing a mask.
Singapore [39]	Wear a mask if you have respiratory symptoms, such as a cough or runny nose.
UK [40]	Wearing a face covering or face mask can reduce the number of particles containing viruses that are released from the mouth and nose of someone who is infected with COVID-19 and other respiratory infections. When you are in close contact with other people, such as in crowded and enclosed spaces, you should wear a face covering or face mask.
Germany [41]	Masks are required when using local and long-distance public transport. A respirator mask (FFP2 or comparable) or medical face mask (mouth-nose protection) must be worn. The obligation to wear a mask does not apply to: children under the age of six; people medically certified as not being able to wear a respirator mask or medical face mask on account of a health disorder, chronic illness or disability; and people who are deaf or hard-of-hearing, the people with whom they communicate, and their accompanying persons. When there is little space in everyday life, wear a mask.

**Table 2:** The structure of the Disposable Surgical Mask and N95 Mask.

Mask Types	Structure	Layers	Materials	Composition	Function
Disposable Surgical Mask	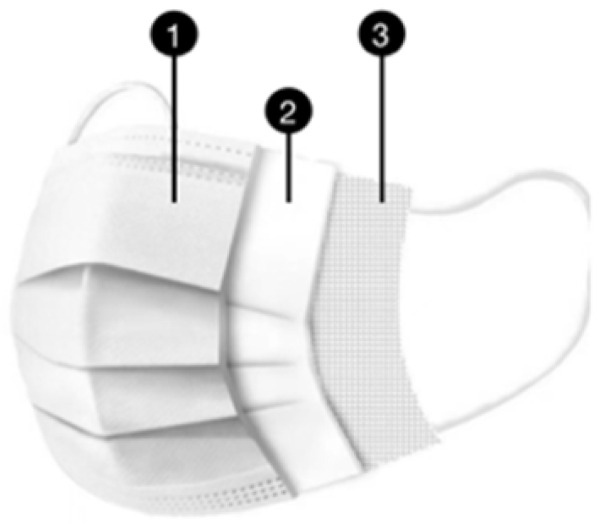	① Outer layer	Non-woven fabric	PP	Block big droplets and particles
		② Middle layer	Melt-blown fabric	PP	Electrostatic filtration
		③ Inner layer	Hot-rolled non-woven fabric	PP	Absorb breathed vapor
N95 Mask	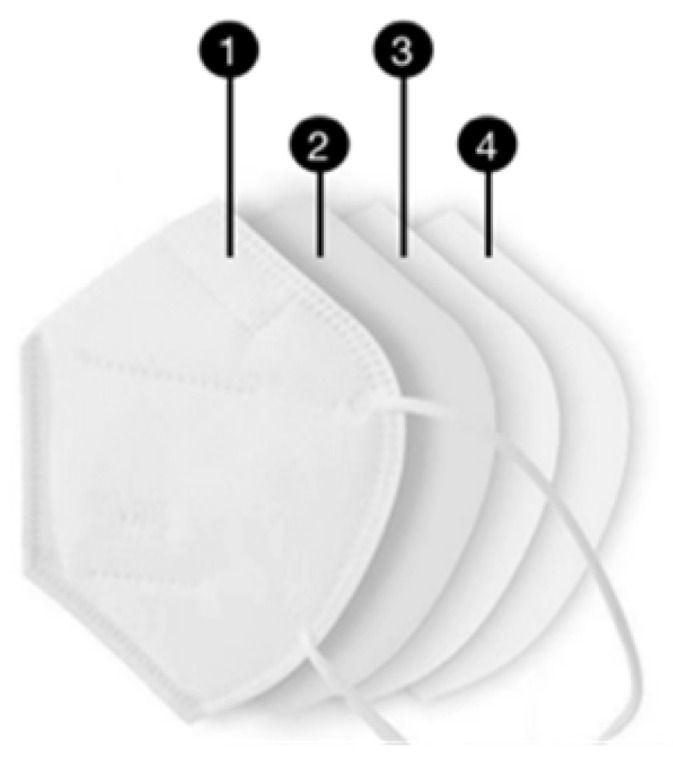	① Outer layer	Non-woven fabric	PP	Filter large particles
		② Filter layer	Melt-blown fabric	PP	Enhance bacterial and fine dust filtration
		③ Support layer	PP cotton	PP	High efficiency electrostatic filtration
		④ Inner layer	Hot-rolled non-woven fabric	PP	Improved comfort in face contact
